# Future Pneumococcal Vaccines: Shifting from Capsular Polysaccharides to Protein-Based Immunogens

**DOI:** 10.3390/vaccines14030208

**Published:** 2026-02-26

**Authors:** Ruodan Zheng, Jiayi Shu, Xingchen Xie, Chen Zhao, Shuye Zhang, Xiaoyan Zhang, Jianqing Xu

**Affiliations:** 1 Institutes of Biomedical Sciences, Fudan University, Shanghai 200032, China; 24211510025@m.fudan.edu.cn (R.Z.); 23111510067@m.fudan.edu.cn (X.X.); 2 Clinical Center of Biotherapy, Zhongshan Hospital, Fudan University, Shanghai 200032, China; shu.jiayi@zs-hospital.sh.cn (J.S.); zhang.shuye@zs-hospital.sh.cn (S.Z.); 3 Shanghai Public Health Clinical Center, Fudan University, Shanghai 201508, China; chen_zhao72@163.com

**Keywords:** *Streptococcus pneumoniae*, vaccine, capsular polysaccharide, subunit, T-cell immunity

## Abstract

*Streptococcus pneumoniae* (*S. pneumoniae*) is a leading cause of pneumonia, meningitis, and sepsis worldwide, posing a major threat to young children and older adults. In China, it is a key pathogen responsible for life-threatening invasive pneumococcal disease (IPD)—including pneumonia, bacteremia, and meningitis—and contributes substantially to hospitalizations and deaths each year. The high disease burden, together with rising antibiotic resistance, underscores the urgent need for more effective strategies for prevention and control. Currently, the most established pneumococcal vaccines include polysaccharide vaccines (e.g., PPV23) and polysaccharide conjugate vaccines (e.g., PCV13), both of which provide effective protection against pneumococcal infections. However, challenges remain, such as the T-cell-independent nature of polysaccharide antigens and inadequate coverage against prevalent strains, which hinder to improve their overall effectiveness. In this review, we trace the progression from pneumococcal pathogenesis to vaccine development. We first outline the mechanisms of colonization, invasion, and key virulence factors, and then critically summarize historical and current vaccine strategies. A systematic literature search was conducted in PubMed and Web of Science (2010–present) using relevant keyword and MeSH combinations. A total of 10,273 articles were identified from PubMed; after removal of duplicates and non-full-text records, 260 research articles were included in the final analysis. Based on this body of evidence, we evaluate emerging approaches toward broadly protective, serotype-independent vaccines and discuss how advances in antigen design, delivery systems, and adjuvants may further optimize next-generation pneumococcal vaccines.

## 1. Introduction

*Streptococcus pneumoniae* (*S. pneumoniae*) remains a major global public health threat, and pneumococcal pneumonia contributes substantially to morbidity and mortality, particularly in developing regions of Asia and Africa. According to the World Health Organization (WHO), pneumonia accounted for approximately 740,180 deaths among children in 2019, with *S. pneumoniae* identified as the leading causative pathogen [1]. In Europe and the United States, the reported incidence of pneumococcal disease ranges from 10 to 100 cases per 100,000 population per year [2].

*S. pneumoniae* is an opportunistic pathogen that commonly co-colonizes the nasopharynx alongside other microbes, including *Moraxella catarrhalis*, *Haemophilus influenzae*, *Neisseria meningitidis*, *Staphylococcus aureus*, and various *Hemolytic streptococci*. Transmission typically occurs via respiratory droplets or direct contact. Although carriage is usually asymptomatic, high-density nasopharyngeal colonization can progress to infection. Individuals at increased risk include young children, older adults, people with underlying medical conditions, and those with compromised immunity [3]. Clinically, pneumococcal disease presents as either non-invasive pneumococcal disease (NIPD)—such as otitis media and sinusitis—or invasive pneumococcal disease (IPD), including community-acquired pneumonia, bacteremia, and meningitis, which can be life-threatening. Both NIPD and IPD impose substantial clinical and economic burdens on patients and healthcare systems [4].

The pneumococcal surface is surrounded by a capsular polysaccharide (CPS) layer. Variation in CPS chemical structure and antigenicity underlies the identification of more than 100 serotypes [5]. Serotypes differ markedly in invasive potential, with serotypes 3, 4, 6B, 9V, 14, 18C, 19F, and 23F historically associated with severe clinical disease. Serotypes sharing the same number belong to the same serogroup and exhibit related capsular structures, and some serogroups (e.g., 6, 10, and 19) show cross-reactivity due to structural similarity.

A growing challenge in pneumococcal disease control is the increasing prevalence of antibiotic resistance. In 2017, *S. pneumoniae* was included in the WHO priority pathogens list for antibiotic research and development. Recent surveillance data from the Centers for Disease Control and Prevention (CDC) indicate that a substantial proportion of pneumococcal infections are resistant to at least one antibiotic. Consequently, the development and deployment of effective pneumococcal vaccines are essential strategies to curb antibiotic resistance and reduce the global burden of disease.

Currently, pneumococcal vaccines fall into two main categories: pneumococcal polysaccharide vaccines (PPVs) and pneumococcal conjugate vaccines (PCVs). The introduction of PCVs into national immunization programs has achieved high protective efficacy in vaccinated children and has been associated with marked declines in pneumonia and IPD. Nevertheless, current vaccines retain important limitations, as discussed below. Developing new, cost-effective vaccines that target conserved pneumococcal antigens to provide broad, serotype-independent protection remains a central goal in pneumococcal vaccine research and development.

Increasing attention has focused on subunit-based immunogens targeting highly conserved pneumococcal proteins. Protein vaccines and nucleic acid vaccines (e.g., mRNA and DNA) are increasingly attracting attention from researchers. For protein vaccines, selecting conserved pneumococcal proteins may help address the issue of serotype replacement. However, key questions regarding their immunogenicity, formulation and combination strategies, breadth of cross-serotype protection, and appropriate evaluation approaches remain to be fully elucidated.

In this review, we first delineate the pathogenic mechanisms of *S. pneumoniae* that shape vaccine design, followed by an assessment of progress in existing pneumococcal vaccine. We searched PubMed and Web of Science (2010–present) using keywords and relevant MeSH terms (e.g., *Streptococcus pneumoniae*, pneumococcal vaccine, next-generation, protein-based, mRNA). In PubMed, broad topic searches yielded 10,273–11,469 records, whereas platform-focused queries narrowed results to 3476 and then 785 records; targeted immunogen/readout queries further refined hits to 326 and 260 records. Web of Science was searched with analogous Topic terms to identify additional relevant records. We organized the selected studies within a pre-defined framework, critically appraised key evidence for rigor, model validity, and consistency, and used this synthesis to survey emerging approaches toward broadly protective, serotype-independent vaccines and, finally, to discuss how advances in delivery systems and adjuvants may further optimize next-generation pneumococcal vaccines.

## 2. Pathogenesis and Immunity of *S. pneumoniae* Infection

### 2.1. Nasopharyngeal Colonization

The transition from asymptomatic pneumococcal carriage to invasive disease typically begins with nasopharyngeal colonization, which depends on a repertoire of bacterial determinants that mediate mucosal adhesion, sustain colonization density, and promote progression toward infection. Capsular polysaccharide (CPS) contributes to this process [2]. Although CPS is not strictly required for initial colonization, its negatively charged surface can electrostatically repel the similarly negatively charged nasal mucus [6], thereby reducing mucus entrapment and clearance and facilitating stable association with the epithelial surface, including the glycocalyx layer.

Beyond CPS, pneumococcal pilus-1, pneumococcal surface protein C (PspC), and pneumococcal surface protein A (PspA) contribute to epithelial adhesion and virulence. The neuraminidase NanA further supports colonization by cleaving terminal sialic acid residues from host mucins, which can enhance bacterial attachment [7]. Additional proteins—including high-temperature requirement protein A (HtrA), subtilisin-like family protein (SFP), cell wall-associated serine protease A (PrtA), and choline-binding protein G (CbpG)—have also been implicated in promoting persistence within the nasopharynx [8]. Moreover, inflammation in the upper respiratory tract can increase epithelial receptivity and thereby enhance *S. pneumoniae* adhesion and colonization [9].

Nasopharyngeal carriage of *S. pneumoniae* is dynamic, with serotypes often replacing one another. In a mouse model, prior colonization with serotype 6B reduced subsequent colonization by 23F after intranasal challenge [10]. By selectively suppressing certain serotypes, vaccination may disrupt these interactions and shift the carriage (and transmission) landscape, so competition and replacement dynamics should be considered in vaccine design.

In addition to serotype turnover at the population level, individual pneumococcal colonies undergo intrinsic changes during colonization. *S. pneumoniae* exhibits phase variation—spontaneous, reversible switching in traits such as metabolism, capsule production, pilus expression, and colony opacity—thereby altering surface properties, colonization efficiency, and virulence. This opaque–transparent opacity switch is observed across most pneumococcal lineages and is linked to coordinated changes in surface architecture, including capsular polysaccharide, cell-wall teichoic acids, and major surface proteins (e.g., PspA and CbpA/PspC).

Functionally, these variants reflect a trade-off between mucosal persistence and invasive potential. Opaque variants generally express more capsule (and less teichoic acid), enhancing bloodstream survival and systemic virulence but often reducing nasopharyngeal colonization; transparent variants express less capsule (and more teichoic acid), improving adhesion and carriage but lowering systemic fitness [11], which is driven by reversible DNA inversions and methylation-dependent switching [12]. Notably, while the capsule can protect pneumococci from mucus-mediated clearance (as mentioned above), it can also hinder adhesion to—and invasion of—non-immune cells [13]. Thus, at mucosal sites exposed to antimicrobial peptides, pneumococci may transiently reduce or shed capsule to promote epithelial interaction; capsule-reduced pneumococci show a 7–8-fold increase in invasion of human lung epithelial cells [14]. The capsule is not a static functional “shield”; rather, pneumococci must make context-dependent trade-offs between its benefits and costs.

### 2.2. Dissemination and Mucosal Penetration

When *S. pneumoniae* colonization reaches a certain density, or when the host’s airway clearance is impaired, the bacteria can break through the mucosal barrier and penetrate deep into the lower respiratory tract, thus entering the lungs. Under normal conditions, the mucociliary apparatus propels inhaled bacteria upward toward the pharynx for expulsion by coughing. However, pneumococcal pneumolysin (Ply) suppresses ciliary beating, while secreted exoglycosidases that degrade mucus (e.g., NanA, BgaA) reduce mucus viscosity, facilitating bacterial transit to the alveoli.

In addition, pneumococci colonizing the nasopharynx may directly breach the mucosal barrier and enter submucosal tissue spaces, lymphatics, and microvasculature, thereby gaining access to the bloodstream and predisposing to invasive pneumococcal disease. Multiple virulence factors contribute to this process:

To counter mucosal immunity, the negatively charged capsule helps evade immune trapping, and the metalloprotease ZmpA can degrade IgA [15]. To disrupt mucosal structures, the surface neuraminidase NanA removes sialic acids from mucus and host cell surfaces, exposing epithelial receptors and promoting adhesion. Ply damages cilia, directly disrupts host cell membranes, and downregulates tight-junction proteins, thereby exposing additional host attachment sites. Moreover, during micro-invasion, bacteria can bind host receptors (e.g., PAFR and pIgR) and hijack host transcytosis pathways to traverse epithelial cells into deeper tissues [16]. Furthermore, two glycolytic enzymes—enolase and glyceraldehyde-3-phosphate dehydrogenase—together with CbpE (also known as Pce), are displayed on the bacterial surface as plasminogen-binding proteins, sequestering and activating host plasminogen to promote accumulation at the pneumococcal surface and enhance penetration through the extracellular matrix [17].

### 2.3. Mechanisms of Systemic Infection

*S. pneumoniae* can infect multiple organs in humans. In addition to the lungs, it can enter the bloodstream and cause bacteremia. Once in the circulation, pneumococci may disseminate to multiple organs, including the spleen and the heart, and can also invade the central nervous system to cause meningitis—although direct spread from the nasopharynx to the brain has also been reported [7].

#### 2.3.1. Pneumonia

When pneumococci disseminate to the lungs and reach the alveoli, pneumonia begins to develop. *S. pneumoniae* releases pneumolysin (Ply), which can directly form pores in alveolar cell membranes, leading to cell death [18], and it also impairs the mucociliary clearance machinery [19]. Once the host immune system detects the bacteria, large amounts of pro-inflammatory mediators are produced, driving neutrophil recruitment into the lungs [20]. To control the infection, vascular permeability increases [21], causing fluid, pus, and red blood cells to flood the alveolar spaces. This compromises gas exchange, and clinically presents with fever, dyspnea, and productive cough.

#### 2.3.2. Lung to Bloodstream (Bacteremia)

In addition to directly traversing the mucosal barrier into the microvasculature, *S. pneumoniae* can also enter the bloodstream via dissemination from the lungs [22]. Within pulmonary mucosal tissues, pneumococci may be carried by dendritic cells during their migration to lymph nodes [23], thereby facilitating systemic spread (e.g., to the blood and the central nervous system). Moreover, pneumococcal factors such as pneumolysin (Ply), neuraminidases, and StkP can damage alveolar epithelial and vascular endothelial cells and/or disrupt tight-junction proteins, promoting direct translocation from the lung into the circulation [24].

Upon entering the bloodstream, *S. pneumoniae* can undergo phase variation toward the opaque phenotype, which is associated with a markedly increased capsule thickness. The negatively charged capsular polysaccharide masks surface antigens and hinders the deposition of complement (C3b) and binding of antibodies, thereby reducing opsonization and impairing recognition and phagocytic clearance by neutrophils and macrophages [25]. In addition, pneumococcal surface protein A (PspA) interferes with complement deposition on the bacterial surface, further promoting immune evasion in the bloodstream [26]. Moreover, pneumococci can recruit host factor H via choline-binding protein A (CbpA); because factor H is a complement regulatory protein that protects host cells from complement-mediated injury, this interaction helps the bacterium evade complement attack by mimicking “self” [27].

In the bloodstream, iron is typically sequestered by lactoferrin or hemoglobin. To ensure survival, *S. pneumoniae* can bind lactoferrin via PspA to counteract its bactericidal activity, and can also acquire iron through interactions with heme and/or hemoglobin [28]. In addition, pneumococci may use hydrogen peroxide to liberate extracellular iron from methemoglobin (Hb-Fe^3+^), thereby increasing access to free iron [29]. Meanwhile, the bacterium preferentially utilizes glucose as a primary carbon source and remodels its metabolic pathways to maintain efficient capsular biosynthesis [15]. Collectively, pneumococcal products can trigger an exaggerated systemic inflammatory response—i.e., sepsis—thereby weakening overall host defenses.

Furthermore, studies have detected increasing numbers of pneumococci within CD169+ macrophages in the spleens of infected humans, suggesting the spleen may serve as a reservoir for bacteremia during infection [30].

#### 2.3.3. Heart (Myocarditis)

In the bloodstream, pneumococci that reach the cardiac microvasculature do not remain confined to the circulation; rather, they can actively traverse vascular endothelial cells and enter the myocardium. The initial invasion of cardiomyocytes is facilitated by specific bacterial surface proteins, with CbpA playing a crucial role alongside host receptors such as the laminin receptor and the platelet-activating factor receptor (PAFR), which are necessary for cellular invasion and the formation of cardiac microlesions [31].

Once within myocardial tissue, the bacteria can rapidly replicate and form microscopic but grossly inapparent microlesions that evade infiltration by neutrophils and other immune cells [31]. In addition, pneumococci have been reported to enter cardiomyocytes, replicate within intracellular vesicles, and adopt a non-purulent biofilm-like state; by rapidly killing resident macrophages, they may further limit immune cell infiltration and increase tolerance to host immune attack [32].

Beyond direct invasion, pneumococci can release two major cytotoxic mediators that directly injure cardiomyocytes. Pneumolysin (Ply), a pore-forming toxin, binds cholesterol in cardiomyocyte membranes to create persistent pores, disrupt ionic homeostasis, and trigger excessive Ca^2+^ influx, leading to osmotic swelling, lysis, and apoptosis [31]. Moreover, bacterially produced hydrogen peroxide induces marked oxidative stress, amplifying damage to surrounding myocardial cells [33].

Host immune responses to pneumococcal infection may also escalate into a dysregulated systemic inflammatory state characterized by excessive release of pro-inflammatory cytokines (e.g., TNF-α and IL-1), which can cause non-specific myocardial injury, depress contractility, and promote vascular leak and shock—collectively reducing coronary perfusion and contributing to myocardial ischemia [34]. Furthermore, pneumococcal infection can potently activate platelets and neutrophils, promoting a prothrombotic milieu that exacerbates ischemic injury; excessive neutrophil extracellular trap (NET) formation may drive microthrombus development, thereby increasing the risk of coronary occlusion and worsening infarction [35].

#### 2.3.4. Meningitis

To invade the brain, *S. pneumoniae* must cross the blood–brain barrier (BBB), which it achieves mainly through three pathways. First, phosphorylcholine (ChoP) moieties on the bacterial surface interact with PAFR expressed on cytokine-activated respiratory epithelial and vascular endothelial cells. The bacterium then hijacks the PAFR recycling pathway to facilitate cellular entry.

**Figure 1:**
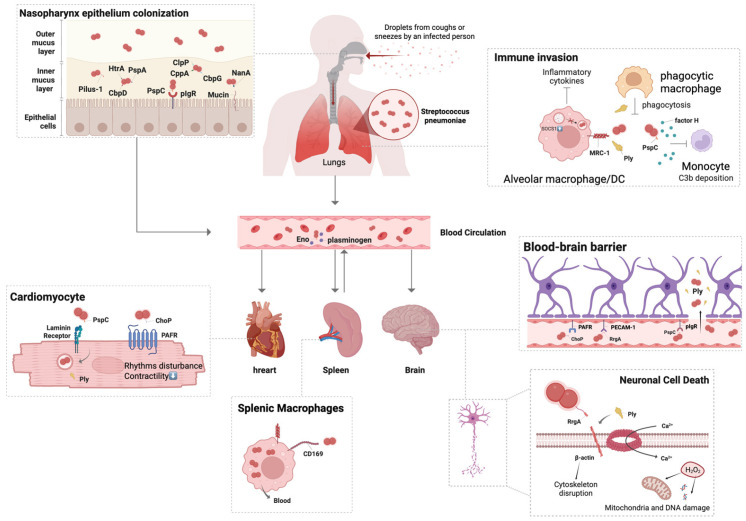
Pneumococcal infection route. *Streptococcus pneumoniae* is transmitted via respiratory droplets and initially colonizes the human nasopharynx. This early colonization is supported by multiple surface factors, including pilus-1, choline-binding protein A (CbpA; also known as PspC), and pneumococcal surface protein A (PspA). Under permissive host or environmental conditions, pneumococci can spread from the nasopharynx to the lower respiratory tract, establish infection in the lungs, and subsequently enter the bloodstream by traversing epithelial and tissue barriers. Following bacteremia, the pathogen may cross endothelial barriers and disseminate to distal organs such as the heart, spleen, and brain. Several pneumococcal virulence determinants—including RrgA, pneumolysin (Ply), and CbpA—have been implicated in facilitating these invasive steps.

Second, as another receptor-mediated route, CbpA binds the laminin receptor (LR) on brain microvascular endothelial cells, thereby promoting BBB traversal during pneumococcal meningitis. Third, the ancillary pilus subunit RrgA, the tip adhesin of pneumococcal pilus-1, interacts with both pIgR and platelet endothelial cell adhesion molecule 1 (PECAM-1) on brain microvascular endothelium [36]. RrgA also binds β-actin on neurons and, together with Ply, contributes to neuronal death [7]. These invasion processes are illustrated in Figure 1.

The third route involves hitchhiking inside infected immune cells. Studies show that inflammatory monocytes (Ly6C+) can carry intracellular pneumococci across the BBB, acting as a “Trojan horse” to establish meningitis. This process is potentiated by host factors like HIV-1 Tat and morphine, which enhance the trafficking of these infected cells into the CNS [37].

As a fourth mechanism, direct physical disruption, the pneumococcal pyruvate oxidase SpxB and α-glycerophosphate oxidase GlpO generate hydrogen peroxide, which can damage the blood–brain barrier (BBB) [38].

Once *Streptococcus pneumoniae* gains access to the cerebrospinal fluid (CSF), it marks a critical turning point in the development of pneumococcal meningitis. Because the CSF contains insufficient complement and antibodies, the bacteria can proliferate almost unrestrained during the early phase. The accumulation of bacterial debris activates microglia in the brain, triggering the release of large amounts of pro-inflammatory mediators (e.g., TNF-α) [39], which in turn drives massive influx of circulating leukocytes—particularly neutrophils—resulting in an inflammatory “storm.” This intense immune response can cause cerebral edema and obstruction of CSF circulation (hydrocephalus), thereby compressing neural tissues. Pneumococcal meningitis is associated with very high mortality, and approximately half of survivors develop long-term neurological sequelae, such as hearing impairment, delayed cognitive development, or epilepsy.

### 2.4. Host Immune Response and Bacterial Immune Evasion

In responding to *S. pneumoniae* infection, the host must maintain a dynamic balance between rapid pathogen clearance (innate immunity) and durable, antigen-specific protection (adaptive immunity). If this balance is disrupted, it can either permit uncontrolled bacterial dissemination or, conversely, drive excessive inflammation (a cytokine storm) that damages host tissues.

During the early stage of pneumococcal infection, host defense is dominated by rapid innate immune responses that limit bacterial expansion. In the lung, resident macrophages recognize pneumococcal components through pattern-recognition receptors, including Toll-like receptors, triggering neutrophil recruitment and phagocytosis [40]. To prevent excessive tissue damage, regulatory mechanisms restrain inflammation: negative regulators such as CYLD dampen NF-κB signaling, while anti-inflammatory cytokines including IL-10 promote timely neutrophil apoptosis and preserve alveolar integrity [41]. Dendritic cells subsequently process bacterial antigens and migrate to draining lymph nodes, where they guide T-cell differentiation. Th17 responses are particularly important, as IL-17A enhances mucosal barrier function and mobilizes additional innate effector cells.

Adaptive immunity then provides antigen-specific clearance and long-term protection. Antibodies against capsular polysaccharides, mainly IgG and IgA, mediate opsonization and facilitate phagocytic uptake [42], while CD4^+^ T cells support durable mucosal immune memory. This coordination enables faster and more controlled responses upon re-exposure [43,44].

Both innate and adaptive immune responses against Streptococcus pneumoniae are highly dependent on the complement system [45]. Antigen–antibody complexes activate the classical pathway via C1 [46], leading to C3b/iC3b deposition and enhanced opsonophagocytosis, whereas membrane attack complex-mediated lysis plays a limited role against Gram-positive bacteria [47].

Pneumococci counteract these mechanisms through virulence factors that interfere with complement activation [48]. The capsule masks surface structures and reduces binding of immunoglobulins, complement components, and C-reactive protein, thereby diminishing C3b deposition and impairing efficient opsonophagocytic killing [46,49].

PspA disrupts complement deposition by binding factor B, thereby inhibiting the formation and/or promoting the dissociation of the alternative pathway C3 convertase [50]. Pneumolysin (Ply) activates the classical complement pathway and can deplete serum opsonizing activity, likely owing to its structural similarity to the Fc portion of IgG. In certain strains, CbpA binds vitronectin-1, which in turn inhibits the classical complement pathway via recruitment of the complement regulator C4b-binding protein (C4BP) [48]. Additional pneumococcal factors that impair opsonophagocytosis include the exoglycosidases NanA, BgaA, and StrH, which likely deglycosylate host glycoproteins involved in complement deposition [51]. Enolase (Eno) binds and activates glyceraldehyde-3-phosphate dehydrogenase (GAPDH), promoting immune evasion by facilitating the degradation of complement components [48]. The autolysin LytB separates daughter cells and enables *S. pneumoniae* to form short chains [52]. Such short-chain pneumococci expose a smaller surface area, which may reduce complement activation and enhance evasion of opsonophagocytic clearance during invasive disease [53].

## 3. Polysaccharide-Based Pneumococcal Vaccine

The global pneumococcal vaccine pipeline is broad and geographically diverse. Licensed pneumococcal vaccines remain focused on the capsular polysaccharides of *S. pneumoniae* and fall into two categories: purified polysaccharide vaccines (PPVs) and polysaccharide conjugate vaccines (PCVs) (Table 1). The highest currently licensed valencies are 23-valent for PPVs and 21-valent for PCVs. Meanwhile, higher-valency PCVs, whole-cell bacterial vaccines, and other next-generation candidates are advancing through clinical trials (Table 2). In China, pneumococcal vaccine use is comparatively limited, with PPV23 and PCV13 being the primary products currently available. Notably, multiple higher-valency vaccines as well as recombinant protein-based candidates are also progressing through clinical trials.

### 3.1. Polysaccharide Vaccines (PPV)

Pneumococcal capsular polysaccharides are immunogenic and form the basis of current pneumococcal vaccines. In the United States, a 14-valent pneumococcal polysaccharide vaccine (PPV) was licensed in 1977 and was later expanded to a 23-valent formulation in 1983. The 23-valent PPV has been used to protect adults at increased risk for invasive pneumococcal disease (IPD) and pneumococcal pneumonia, and it has also been administered to older adults, including those aged ≥ 65 years. [54]. A five-year prospective study in Shanghai followed adults aged ≥ 60 years to assess the durability of antibody responses elicited by the 23-valent pneumococcal polysaccharide vaccine (PPV23). The investigators reported that antibody levels against most serotypes remained relatively high over the entire five-year follow-up period [55]. In a Danish study assessing PPV23 effectiveness against invasive pneumococcal disease (IPD), vaccinated adults aged ≥ 65 years showed protection against overall IPD (vaccine effectiveness [VE] 32%) and IPD caused by PPV23 serotypes (VE 41%), with particularly high effectiveness against serotype 8 (VE 62%) and serotype 22F (VE 88%). Protection was also observed against IPD caused by PPV23 serotypes not included in PCV15 (VE 50%) and against PCV20 serotypes (VE 61%). In contrast, PPV23 did not protect against serotype 3 IPD (VE −17%) [56].

A major limitation of pneumococcal polysaccharide vaccines (PPVs) is that purified capsular polysaccharides are relatively weak immunogens because they are thymus-independent antigens that primarily elicit T cell-independent responses. As a result, they generate limited affinity maturation and little to no immunological memory, leading to antibody responses that wane over time. Consequently, PPVs are ineffective in infants younger than two years of age [57]. In addition, the effectiveness of PPV23 against non-invasive pneumococcal pneumonia remains debated, as many studies have not consistently demonstrated protection against non-bacteremic pneumonia or reductions in mortality. Evidence supporting a protective benefit of PPV23 in populations at high risk for HIV infection is also insufficient [58]. Moreover, vaccine effectiveness declines with time [59], with protection generally lasting less than five years. Collectively, these limitations highlight the need for alternatives to purified capsular polysaccharide vaccines to better protect vulnerable populations.

### 3.2. Polysaccharide Conjugate Vaccines (PCV)

Because protein antigens elicit antibody responses early in life and, unlike polysaccharides, are processed as T cell-dependent antigens that efficiently drive class switching and immunological memory, pneumococcal conjugate vaccines (PCVs) were developed by chemically linking capsular polysaccharides to protein carriers [54]. Common carrier proteins include CRM197, tetanus toxoid, and diphtheria toxoid. Although higher-valent formulations (up to 21-valent, PCV21) have been developed, PCV13 remains among the most widely used PCVs globally.

Compared with pneumococcal polysaccharide vaccines (PPVs), PCVs generally induce stronger and more durable immune responses, generating higher levels of functional antibody that can limit pneumococcal adhesion at mucosal surfaces. For example, the pneumococcal zinc metalloprotease ZmpA (IgA1 protease) cleaves the hinge region of human IgA1; however, PCV vaccination elicits high concentrations of protease-resistant IgG that can transudate to mucosal sites [60], thereby interfering with colonization and providing serotype-specific protection [61].

In addition to direct protection in vaccinated individuals—primarily children and older adults—PCVs can confer herd effects by reducing carriage of vaccine serotypes in children, including lineages commonly associated with antimicrobial resistance, thereby decreasing transmission at the population level [54]. Reported PCV effectiveness is high, commonly in the range of 70–85% [62], and Zhang’s group reported that PCV13 could prevent > 90% of invasive infections caused by vaccine serotypes [63]. Nevertheless, several challenges remain.

First, serotype replacement continues to be a major concern. Because both PCVs and PPVs target defined capsular serotypes, declines in vaccine serotypes can be accompanied by increases in non-vaccine serotypes, driving the ongoing need for higher-valent formulations. Multiple factors influence replacement dynamics, including pre-vaccine serotype prevalence, antibiotic selection pressure, clonal lineage shifts, environmental factors (e.g., daycare attendance, household crowding and siblings), vaccination policies, and serotype-specific invasiveness [1].

Second, the move toward high-valent PCVs introduces additional complexity. To broaden coverage, vaccines with 15–20+ serotypes have been introduced; Dr. Ryman’s group evaluated serotype-specific effectiveness of V114 (PCV15) and PCV20 against pediatric invasive pneumococcal disease and reported that V114 maintained higher effectiveness than PCV20 for most serotypes shared with PCV7 and PCV13 [64]. One proposed explanation is carrier-induced epitope suppression (CIES), whereby increasing the amount of carrier protein relative to polysaccharide can dampen polysaccharide-specific antibody responses and reduce effective titers [65].

In parallel, expanded use of polysaccharide-based vaccines has been accompanied by increased recognition of infections caused by non-encapsulated *S. pneumoniae* (NESp), which are not covered by capsule-based formulations. NESp express pneumococcal surface protein Korea (PspK; previously termed non-typeable pneumococcal surface protein, NspA), encoded within the capsule synthesis locus; PspK is structurally related to pneumococcal surface protein C (PspC) and is thought to enhance adhesion and nasopharyngeal colonization, partially compensating for the absence of a capsule [66].

Finally, PCVs remain costly for national immunization programs because of complex manufacturing and quality-control requirements, contributing to lower uptake in many low- and middle-income settings. In addition, multi-dose schedules (“3 + 1”,”2 + 1”regimen) [67] can reduce compliance and limit coverage. Taken together, while PCVs have substantially reduced vaccine-type disease, they have not fully resolved serotype replacement and leave important gaps in affordability and breadth of protection [68,69].

### 3.3. High-Valency and Alternative PCV Architectures

As one of the most established pneumococcal vaccine platforms, PCVs have substantially reduced pneumococcal disease burden, despite the limitations noted above. Accordingly, higher-valency PCVs continue to be pursued to broaden serotype coverage and mitigate serotype replacement. In the United States, PCV21 (e.g.,V116) and PCV20 (e.g., Prevnar 20) have been incorporated into the U.S. Centers for Disease Control and Prevention (CDC) recommendations for adults aged ≥ 65 [70], and PCV24 candidates are also under development.

To tackle the problem of CIES, several pneumococcal vaccines have introduced innovations based on PCV technology. Vaxcyte’s Vax series of polysaccharide conjugate vaccines employ click chemistry to specifically and covalently attach the polysaccharide antigen to a proprietary detoxified diphtheria carrier protein (eCRM) through non-natural amino acid (nnAA) sites [71]. VAX-31 (31-valent PCV) is being evaluated in a Phase 2 infant dose-finding study, while robust opsonophagocytic activity (OPA) responses across all 31 serotypes at all doses tested have been reported in a Phase 1/2 study in adults (as Table 2). Unlike conventional random conjugation, Vaxcyte’s platform uses an engineered CRM197 carrier (eCRM) containing multiple genetically encoded non-natural amino acids (nnAAs) to enable site-specific polysaccharide conjugation, which is designed to preserve key T-cell epitopes and reduce conjugation heterogeneity [71,72]. By increasing the polysaccharide:protein ratio and limiting carrier-related interference, this strategy is intended to help mitigate issues such as CIES; however, direct pharmacodynamic confirmation of reduced effective carrier load in humans would require dedicated studies.

Synflorix (PCV10) adopts a mixed-carrier design—different capsular polysaccharides are conjugated to protein D, tetanus toxoid (TT), or diphtheria toxoid (DT). By distributing serotypes across multiple carriers, Synflorix reduces repeated competition for the same carrier-specific T-cell help, which may lessen carrier dominance as valency and schedule complexity increase [73].

Building on prior work in PCV technology, studies from the Thanawastien group challenge the conventional assumption that covalent coupling of polysaccharides to carrier proteins is strictly required to convert polysaccharides into T cell-dependent immunogens [74]. Their approach, termed PCMV, encapsulates polysaccharides within a cross-linked protein matrix, which is sufficient to elicit robust immune response while preserving key functional attributes of traditional conjugate vaccines and minimizing unnecessary alterations of carbohydrate epitopes [69]. Compared with conventional PCVs, the PCMV manufacturing workflow is markedly streamlined. For example, a typical 13-valent conjugate vaccine entails 13 separate chemical activation and/or conjugation reactions, whereas PCMV can be produced through a single chemical cross-linking step with fewer processing stages. This simplified process may facilitate incorporation of additional polysaccharides, enabling higher-valency formulations with broader serotype coverage. If ongoing studies further validate PCMV stability and immunogenicity, its versatility and manufacturability could support scalable vaccine production against encapsulated pathogens and potentially improve access in developing countries.

### 3.4. Synthetic Oligosaccharides as Defined Antigens

Despite the clinical success of pneumococcal conjugate vaccines (PCVs), traditional polysaccharide–protein conjugates present inherent limitations, including high manufacturing costs, batch-to-batch variability, structural heterogeneity of extracted capsular polysaccharides, and challenges in achieving precise and reproducible conjugation under GMP conditions. The natural polysaccharides used in current PCVs are biologically derived and often contain impurities or undefined structural variations, which may affect consistency and scalability.

To overcome these limitations, synthetic carbohydrate approaches have emerged as a promising alternative. Advances in carbohydrate chemistry have enabled the total synthesis of structurally defined capsular polysaccharides, as demonstrated for serotype 14 [75]. More importantly, current efforts focus on identifying minimal antigenic oligosaccharide epitopes that retain immunogenicity while offering superior structural homogeneity. These defined oligosaccharides can be conjugated to carrier proteins to generate so-called third-generation carbohydrate vaccines, characterized by precise composition, improved reproducibility, and potentially enhanced safety profiles [76,77].

Several synthetic oligosaccharide fragments representing key antigenic determinants of pneumococcal capsular polysaccharides have been developed and evaluated as glycoconjugate vaccine candidates, showing encouraging immunogenicity in preclinical studies [78]. Such structurally defined constructs provide better control over epitope presentation and may reduce off-target immune responses associated with heterogeneous polysaccharide preparations. Collectively, synthetic carbohydrate-based vaccines represent a rational evolution of conjugate technology and an important direction in the future development of pneumococcal subunit vaccines.

## 4. Future Pneumococcal Vaccines: Transition to Protein-Based

### 4.1. Protein Vaccine

Protein pneumococcal vaccines offer a promising strategy to overcome the limitations of PPVs and PCVs. Unlike capsular polysaccharides, which vary significantly across serotypes, some pneumococcal proteins are highly conserved, making them attractive targets for broad-spectrum vaccines [79]. This approach is motivated in part by the persistent challenge of serotype substitution under capsule-focused vaccine pressure [80].

Representative antigens span distinct functional categories: toxins (detoxified pneumolysin, Ply) to provide anti-toxin protection [81]; adhesins and choline-binding proteins such as CbpA that are highly prevalent and strongly immunogenic during natural colonization [57]; and surface-exposed proteins including the Pht family (e.g., PhtD/PhtE), which can elicit cross-reactive antibodies and have been linked to colonization and disease-associated phenotypes [81]. In addition, autolysins (e.g., LytA/LytB) [82,83,84] and other conserved surface factors [79,85] have been explored as vaccine targets because they contribute to host interaction and immune evasion.

Immunogenicity data further support prioritization of these targets: in young children, naturally acquired antibody and memory responses have been observed against several candidate proteins (including PhtD/PhtE, PcpA, Ply, and LytB), consistent with their in vivo expression during colonization and infection [81]. Among the most widely studied antigens, PspA is nearly universal and highly immunogenic [86]. Beyond these classical candidates, additional enzymes and lipoproteins are being investigated—for example rPgdA, which reduced pulmonary invasion in preclinical studies [87], and lipoproteins such as PsaA (and others including MetQ and DacB) that are surface-exposed and conserved, immunogenic, and can activate antigen-presenting cells, making them attractive components for next-generation multicomponent formulations [88,89].

However, because pneumococcal surface proteins are sequence-variable, no single protein antigen is conserved enough to provide standalone protection against *S. pneumoniae* [90], as also shown in preclinical models [91]. Accordingly, the field is shifting toward polyvalent protein designs—combining multiple antigens (mixtures, fusion proteins, or nanoparticle formats) to target complementary mechanisms such as anti-toxin activity, anti-adhesion, immune-evasion interference, and T cell–dependent mucosal clearance [90]. For example, multivalent fusion antigens (e.g., YAPO/YAPL) can elicit broader immunity than single proteins [92]; Milani’s group [93] fused an N-terminal fragment of PspA with a detoxified pneumolysin derivative (PlD) inducing cross-protective immunity in mice, although identifying optimal antigen combinations still requires extensive screening [91]. By engaging T cell help, protein antigens can induce Th17-skewed mucosal immunity that promotes neutrophil recruitment and improves colonization control [94], while also supporting more durable memory than T cell-independent polysaccharides and benefiting from nanoparticle-based repetitive antigen display to boost B-cell and antibody responses [95].

Protein vaccines may scale more readily than high-valency PCVs because a small set of conserved protein antigens could replace the separate purification and conjugation steps required for many distinct polysaccharides, reducing manufacturing complexity and cost [96]. Once a recombinant protein “platform” is established, it can be extended to additional antigens to speed multivalent development and large-scale supply [97].

Nevertheless, while these advantages are conceptually compelling, robust correlates of protection and standardized evaluation frameworks for protein vaccines remain less established than those for PCVs (e.g., serum IgG concentrations and OPA titers), and comprehensive clinical validation is still needed. This is largely because protein antigens can protect via multiple, overlapping mechanisms—such as toxin neutralization/anti-adhesion antibodies, complement-dependent activity, and Th17/IL-17A–mediated mucosal clearance—so a single assay (including OPA) may not capture the full spectrum of protective immunity [98].

Therefore, protein-vaccine evaluation typically relies on a combined set of readouts, including in vitro binding assays [99] and serum protection associations with anti-protein antibodies [100], functional assays such as Ply neutralization/hemolysis inhibition [101] and complement deposition [99,102], alongside cellular immunity metrics like IL-17A responses [94,103]. These are commonly complemented by in vivo efficacy endpoints, including survival in lethal challenge/sepsis models [104], reduced lung bacterial burden in pneumonia models [105], and decreased nasopharyngeal colonization in carriage models [106].

Overall, while additional studies are needed to fully establish broadly applicable correlates of protection and to validate efficacy across diverse clinical settings, the accumulating preclinical and early clinical evidence supports substantial developmental potential for protein pneumococcal vaccines. Notably, protection has been demonstrated using detoxified Ply antigens [107], fusion proteins such as ΔA146Ply–SP0148 that protect against lethal and pulmonary infection in mice [108], and combinations such as pneumolysin plus a CbpA-derived fragment that elicit cross-serotype protection in mouse model [109]. Collectively, these findings reinforce the emerging consensus that polyvalent, mechanism-complementary protein vaccines tend to be a leading strategy toward broad, serotype-independent pneumococcal protection [90].

### 4.2. Pneumococcal Proteins as Carriers in Conjugate Vaccines

As discussed above, polysaccharide–protein conjugate vaccines elicit strong, well-established protection, but this protection is largely restricted to the capsular serotypes included in the formulation. In contrast, protein vaccines can induce antibodies that recognize pneumococci across many serotypes, although the extent of protective efficacy remains to be confirmed. To combine these advantages, several groups have explored using pneumococcal proteins as carrier antigens in conjugate designs to preserve robust anti-capsular immunity while adding broader anti-protein responses.

Regarding a vaccine candidate formulated by da Silva et al. [110] that contains the protein PspA conjugated to the capsular polysaccharide serotype 14, immunized mice showed high IgG antibodies with opsonophagocytic activity but more importantly, the immunization protected from *S. pneumoniae* serotype 6. Another study [111] developed a vaccine where the protein PspA was conjugated to the capsular polysaccharide serotype 6B. Immunization of mice with this formulation produced IgG antibodies against both the capsular polysaccharide and the protein PspA.

Detoxified pneumolysin (Ply) or PspA were used as carrier proteins to conjugate synthetic oligosaccharide antigens targeting pneumococcal serotype 2 or 3 [112]. Proof-of-concept experiments in mouse and pig models showed that these synthetic glycoconjugates can inhibit nasopharyngeal colonization, reduce bacterial burden, and alleviate disease severity after challenge [112]. In parallel, the MAPS-based candidate ASP3772—comprising 24 biotinylated pneumococcal polysaccharides linked to two surface proteins (SP1500 and SP0785)—was generally well tolerated in adult and older-adult cohorts (e.g., fatigue, headache, myalgia) and induced functional antibodies to all included serotypes, alongside increased SP1500/SP0785-specific Th17 responses and higher IL-17 production in stimulated PBMCs [113].

Collectively, these approaches provide additional routes to improve next-generation pneumococcal vaccines by integrating serotype-specific anti-capsular immunity with broader anti-protein responses, potentially enhancing resilience against serotype replacement.

### 4.3. Nucleic Acid Pneumococcal Vaccines

The COVID-19 pandemic greatly accelerated the maturation and global adoption of mRNA vaccine technology, underscoring its exceptional development speed and platform-based flexibility [114,115]. As a result, mRNA approaches are now being actively explored across a broad range of bacterial vaccine programs, including for respiratory pathogens, where rapid iteration and modular antigen design are particularly advantageous. Mechanistically, mRNA is non-integrating and transient, and can be rationally engineered to drive in situ expression of antigens that closely resemble native proteins, while avoiding genome integration concerns [115]. Prior to COVID-19, multiple groups had already advanced mRNA platforms in oncology and viral vaccines (e.g., influenza), providing a foundation for broader infectious-disease applications [116,117,118].

Accumulating evidence indicates that mRNA design itself is a decisive lever for antibacterial efficacy—particularly the choice of protective antigens and how they are encoded. Several construct-level strategies that are especially relevant for bacteria, including (i) rational selection of surface proteins/toxins as immunogens; (ii) multivalent or multiepitope designs to broaden coverage and tune humoral–cellular immunity; and (iii) optimization of antigen sequence architecture and signal elements to ensure correct expression and immune focusing in eukaryotic cells [119].

Building on this framework, pioneering studies of an mRNA vaccine against Yersinia pestis [120] demonstrate the feasibility of eliciting protective antibacterial immunity. In another bacterial example, a multicomponent mRNA-LNP vaccine encoding five conserved Group A Streptococcus antigens (Combo#5) elicited broad adaptive immune responses (including T-cell and B-cell memory features) and protected mice in challenge models, reinforcing the value of multivalent antigen selection and encoding in bacterial settings [121]. Also, recent comprehensive reviews highlighted summarizing the mRNA vaccine development progress of diverse pathogens (e.g., *M. tuberculosis*, *P. aeruginosa*, *L. monocytogenes*, *S. pyogenes*, *B. burgdorferi*) and emphasized that bacterial mRNA vaccine development is increasingly feasible [119].

Together, these advances support mRNA vaccination as a promising and rapidly adaptable strategy for pneumococcal prevention, and interest in this direction is rapidly growing. For example, a recent study reported a prophylactic mRNA vaccine encoding conserved pneumococcal protein antigens —histidine triad protein D (PhtD) and pneumolysin (Ply)—that was rationally designed to promote antigen expression and to elicit both systemic and mucosal immunity. This vaccine conferred cross-serotype protection and produced measurable reductions in colonization as well as lethal infection in a mouse model [122].

Collectively, these findings position mRNA vaccination as a promising next-generation strategy for pneumococcal prevention. At the same time, sustained progress will likely depend on continued optimization of antigen selection and mRNA construct design, together with delivery and adjuvant approaches that enhance the magnitude, durability, and mucosal breadth of protection.

## 5. Delivery and Adjuvants

Traditional pneumococcal conjugate vaccines (PCVs) are administered by injection and primarily induce systemic immunity, with limited ability to elicit classical mucosal responses such as high levels of secretory IgA [123]. Nevertheless, their effectiveness in reducing nasopharyngeal carriage and transmission is well established [124]. Because *S. pneumoniae* spreads via respiratory droplets and colonizes the upper airway [7], intranasal vaccination remains an attractive strategy to enhance local mucosal immunity at the portal of entry. In principle, intranasal delivery could further strengthen protection by boosting site-specific immune responses, limiting colonization, and improving acceptability through needle-free administration [125].

Abera Bioscience is developing an intranasal pneumococcal vaccine candidate, Ab-01.12. This vaccine leverages a universal Gram-negative bacterial outer membrane vesicle (OMV) platform—non-infectious particles naturally released by bacteria that can enhance immune responses [126]. Delivered as a nasal spray, Ab-01.12 is intended to induce robust mucosal immunity at the site of initial pneumococcal colonization. In a complementary approach, Nakahashi-Ouchi’s study [127] developed an adjuvant-free, cationic nanogel-based nasal vaccine (cationic cholesteryl pullulan, cCHP) formulated with a trivalent PspA antigen, which suppressed lung inflammation and markedly reduced lung bacterial burdens in rhesus monkeys following intratracheal pneumococcal challenge.

With respect to adjuvant strategies, the invariant natural killer T (iNKT) cell agonist α-galactosylceramide (α-GalCer) has been widely explored in preclinical studies. Wei Peng’s study [128] reported an intranasal formulation (LipoCPS12F & α-GalCer) in which serotype 12F capsular polysaccharide and α-GalCer were co-encapsulated in cationic liposomes; in mice, this approach activated iNKT cells, promoted B-cell maturation, and conferred protective immunity, supported by functional in vitro assays. Rodrigues’ research [129] developed spray-dried lipid/polymer nanocomposite particles (LP/NCMPs) composed of dipalmitoylphosphatidylcholine (DPPC) and DC-Chol, incorporating α-GalCer as an adjuvant and encapsulating PspA1 and PspA4Pro within alginate-stabilized particles; immunization provided full protection against *S. pneumoniae* strains expressing PspA families 1 and 2, and CD4^+^ resident memory T cells were detected in the lungs of surviving animals, consistent with durable mucosal protection.

Beyond α-GalCer–based systems, intranasal administration of Corynebacterium pseudodiphtheriticum 090104 (Cp) or Cp-derived bacterial-like particles (BLPs) has also been investigated as a mucosal adjuvant strategy to enhance pneumococcal vaccine immunogenicity. Ramiro Ortiz Moyano’s study [130] showed that co-administration of the pneumococcal fusion antigen PSPF (PsaA–Spr1875–PspA–FliC) with Cp or Cp-derived BLPs significantly augmented immune responses in a mouse model.

The incorporation of CpG oligodeoxynucleotide (ODN) adjuvants into pneumococcal glycoconjugate vaccines has been reported to enhance protective immunity against pneumococcal infection [131]. Flt3 ligand (FL)—a hematopoietic growth factor that signals through the Fms-like tyrosine kinase 3 receptor (Flt3/Flk2)—has also been explored as a potent mucosal adjuvant for pneumococcal immunization. In mouse models, co-delivery of unmethylated CpG ODN together with a plasmid encoding FL (pFL) and pneumococcal antigens has been shown to induce protective immunity [132]. In addition, a nasal pFL-based adjuvant system formulated with phosphorylcholine–keyhole limpet hemocyanin (PC–KLH) has been reported to confer complete protection against *S. pneumoniae* nasal colonization.

More recently, Kosuke Fujimoto and Satoshi Uematsu [133] developed a vaccine platform that combines emulsified curdlan with CpG ODN, which elicited robust antigen-specific systemic and mucosal immune responses to pneumococcal antigens in mice.

## 6. Conclusions and Discussion

*S.pneumoniae* is commonly carried in the nasopharynx of children in China and can cause a broad spectrum of serious diseases, including pneumonia and meningitis, with some cases resulting in long-term or lifelong sequelae. Beyond the substantial health impact, pneumococcal infections also impose considerable economic burdens on families. In response to rising antimicrobial resistance and the need to prevent severe invasive pneumococcal disease (IPD), multiple pneumococcal vaccines have been developed and introduced. At present, PCV13 and PPV23 are among the most widely used formulations and provide meaningful, although incomplete, protection. However, PPV23 does not elicit adequate immune responses in children younger than two years and generally provides protection that wanes within approximately five years. Although PCVs are highly immunogenic in young children, their overall fiscal burden in immunization programs can be substantial—driven not only by the per-dose price, but also by the multi-dose infant schedules required to fully immunize each child (e.g., 2 + 1/3 + 0 or 3 + 1 regimens), which increases the total cost per vaccinated child even when procurement prices are discounted [134,135]. In addition, PCVs entail greater manufacturing complexity than PPVs because each polysaccharide must be chemically conjugated to a carrier protein, increasing production and quality-control demands. Finally, both PCVs and PPVs are affected by serotype replacement, with non-vaccine serotypes increasing in prevalence over time.

To address the limitations of PPVs and existing PCVs, numerous higher-valent PCVs and next-generation pneumococcal vaccine candidates are currently in development or clinical evaluation. Much of this effort has focused on expanding valency to include emerging non-vaccine serotypes. However, increasing valency alone is unlikely to fully overcome fundamental constraints, including serotype-restricted protection, limited induction of mucosal immunity, the creation of ecological space that may favor non-encapsulated strains, and CIES. Moreover, as valency rises, manufacturing becomes more complex and the potential for immune interference increases. Consequently, a central challenge for the field is to identify broadly protective antigens that are not limited by serotype and can elicit cross-protective immunity, while maintaining an optimal balance among breadth of coverage, minimized CIES, and manufacturing feasibility.

Achieving broad, serotype-independent protection with non-capsular antigens remains challenging. Beyond capsular polysaccharides, multiple pneumococcal surface proteins have been investigated as alternative vaccine targets; however, many are genetically diverse and exhibit limited sequence conservation. Even widely expressed antigens such as PspA and PspC can vary substantially in sequence and structure across serotypes—and even among strains within the same serotype—leading to heterogeneous antigenicity and immunogenicity. As a result, identifying regions that are sufficiently conserved, surface-exposed, and consistently immunogenic remains a key bottleneck for protein vaccine design.

Importantly, while protein antigens offer the promise of broader coverage, they face distinct hurdles, including the likely need for multi-component formulations to achieve reliable protection across the global strain repertoire. This requirement represents a major developmental challenge, in contrast to the more established, serotype-specific correlate of protection used for PCVs. Consistent with this, several protein-based candidates have shown limited advancement in clinical development to date, and their protective efficacy across diverse serotypes remains incompletely defined.

To address these challenges and accelerate next-generation immunogen design, bioinformatics and AI-driven approaches may provide valuable tools. Integrating comparative genomics, protein structure modeling, and computational epitope mapping can enable systematic identification of conserved and immunologically accessible regions across diverse *S. pneumoniae* strains, supporting the rational design of optimized B-cell and T-cell immunogens.

Looking ahead, future vaccine development should also prioritize equity and accessibility. In addition to improving breadth beyond serotype-specific targets, advances in dose-sparing strategies, adjuvant selection, and simplified delivery could expand coverage across diverse populations. Finally, strengthened genomic surveillance and rigorous cost-effectiveness evaluations will be essential to guide adaptive vaccination policies and improve global pneumococcal disease control.

## Figures and Tables

**Table 1:** Pneumococcal vaccines used globally.

Type (Trade Name)	Company	Population	Coverage of Serotypes	Vaccination Procedure	Licensed Region
23-valent polysaccharide vaccine	Beijing Zhifei lvzhu (Beijing, China)	≥2 years of age	1, 2, 3, 4, 5, 6B, 7F, 8, 9N, 9V, 10A, 11A, 12F, 14, 15B, 17F, 18C, 19A, 19F, 20, 22F, 23F and 33F	1 dose, 0.5 mL IM; revaccination for high-risk	China
23-valent polysaccharide vaccine	Sinovac (Beijing, China)	≥2 years of age	1, 2, 3, 4, 5, 6B, 7F, 8, 9N, 9V, 10A, 11A, 12F, 14, 15B, 17F, 18C, 19A, 19F, 20, 22F, 23F and 33F	1 dose, 0.5 mL IM; revaccination for high-risk	China
23-valent polysaccharide vaccine	Beijing Minhai Biotechnology (Beijing, China)	≥2 years of age	1, 2, 3, 4, 5, 6B, 7F, 8, 9N, 9V, 10A, 11A, 12F, 14, 15B, 17F, 18C, 19A, 19F, 20, 22F, 23F and 33F	1 dose, 0.5 mL IM; revaccination for high-risk	China
23-valent polysaccharide vaccine	Chengdu Institute of Biological Products (Chengdu, China)	≥2 years of age	1, 2, 3, 4, 5, 6B, 7F, 8, 9N, 9V, 10A, 11A, 12F, 14, 15B, 17F, 18C, 19A, 19F, 20, 22F, 23F and 33F	1 dose, 0.5 mL IM; revaccination for high-risk	China
23-valent polysaccharide vaccine	Walvax Biotechnology (Kunming, China)	≥2 years of age	1, 2, 3, 4, 5, 6B, 7F, 8, 9N, 9V, 10A, 11A, 12F, 14, 15B, 17F, 18C, 19A, 19F, 20, 22F, 23F and 33F	1 dose, 0.5 mL IM; revaccination for high-risk	China
23-valent polysaccharide vaccine (Pneumovax23^®^)	Merck Sharp & Dohme (Rahway, NJ, USA)	≥50 years of age ≥1 year of age in high-risk population	1, 2, 3, 4, 5, 6B, 7F, 8, 9N, 9V, 10A, 11A, 12F, 14, 15B, 17F, 18C, 19A, 19F, 20, 22F, 23F and 33F	Label dosing is single 0.5 mL dose (IM/SC);	China
13-valent polysaccharide conjugate vaccine (Prevenar13)	Pfizer (New York, NY, USA)	6 weeks to 15 months of age	1, 3, 4, 5, 6A, 6B, 7F, 9V, 14, 18C, 19A, 19F and 23F	4 doses (2, 4, 6 months + booster at 12–15 months), 0.5 mL IM	China
13-valent polysaccharide conjugate vaccine	Beijing Minhai (Beijing, China)	6 weeks to 5 years of age	1, 5, 6A, 9V, 19A, 19F, 23F (bound to tetanus toxin protein) 3, 4, 6B, 7F, 14, 18C (bound to diphtheria toxoid protein)	Follow local label/NIP (commonly 3 + 1 or 2 + 1)	China
13-valent polysaccharide conjugate vaccine	Walvax Biotechnology (Kunming, China)	6 weeks to 5 years of age	1, 3, 4, 5, 6A, 6B, 7F, 9V, 14, 18C, 19A, 19F and 23F	Follow local label/NIP (commonly 3 + 1 or 2 + 1)	China
21-valent polysaccharide conjugate vaccine (V116)	Merck & Co(Rahway, NJ, USA)	≥18 years of age	3, 6A, 7F, 8, 9N, 10A, 11A, 12F, 15A, 15C, 16F, 17F, 19A, 20A, 22F, 23A, 23B, 24F, 31, 33F and 35B	Single 0.5 mL IM dose (adults, one-dose schedule)	United States (FDA); European Union (EC/EMA)
20-valent polysaccharide conjugate vaccine (Prevnar 20)	Pfizer (New York, NY, USA)	≥6 weeks of age	1, 3, 4, 5, 6A, 6B, 7F, 8, 9V, 10A, 11A, 12F, 14, 15B, 18C, 19A, 19F, 22F, 23F and 33F	Adults: typically single 0.5 mL IM dose; pediatrics per local label/NIP	United States (FDA); European Union (EC/EMA) and other regions
15-valent polysaccharide conjugate vaccine (VAXNUEVANCE)	Merck & Co (Rahway, NJ, USA)	≥6 weeks of age	1, 3, 4, 5, 6A, 6B, 7F, 9V, 14, 18C, 19A, 19F, 22F, 23F, 33F	Infants: commonly 4-dose series (2, 4, 6 months + booster at 12–15 months) or per local schedule; adults: often single dose depending on policy	United States (FDA); European Union (EC/EMA)
10-valent polysaccharide conjugate vaccine (Synflorix)	GSK (London, UK)	6 weeks to 5 years of age	1, 4, 5, 6B, 7F, 9V, 14, 18C, 19F, 23F	Commonly 3 + 1 or 2 + 1 from 6 weeks of age (program-dependent)	European Union
10-valent polysaccharide conjugate vaccine (Pneumosil)	Serum Institute of India (Pune, India)	6 weeks to 2 years of age	1, 4, 5, 6A, 6B, 7F, 9V, 14, 18C, 19F, 23F	Program schedules typically 2 + 1 or 3 + 0 (EPI-dependent), 0.5 mL per dose	WHO Prequalified (used across multiple countries via procurement programs)

**Table 2:** Pneumococcal vaccines in clinical trials.

Type (Trade Name)	Company	Publishing Time	Status	Coverage of Serotypes	Locations	ClinicalTrials.gov ID
26-valent polysaccharide conjugate vaccine	Beijing Zhifei lvzhu	27 January 2025	Phase I/II (Child, Adult, Older Adult) ≥2 months of age, enrolling by invitation	1, 2, 3, 4, 5, 6A, 6B, 7F, 8, 9N, 9V, 10A, 11A, 12F, 14, 15B, 17F, 18C, 19A, 19F, 20, 22F, 23F, 24F, 33F and 35B	China	NCT06703203
24-valent polysaccharide conjugate vaccine	Sinovac	24 December 2025; 28 January 2026; 16 April 2025	Phase II (pediatric), recruiting; Phase 1 (Child, Adult, Older Adult), completed	1, 2, 3, 4, 5, 6A, 6B, 7F, 8, 9N, 9V, 10A, 11A, 12F, 14, 15B, 17F, 18C, 19A, 19F, 20, 22F, 23F and 33F	China	NCT07300644; NCT06550830; NCT06474377
24-valent polysaccharide conjugate vaccine	Shanghai Reinovax	15 April 2024	Phase II (adult), active, not recruiting	1, 2, 3, 4, 5, 6A, 6B, 7F, 8, 9N, 9V, 10A, 11A, 12F, 14, 15B, 17F, 18C, 19A, 19F, 20, 22F, 23F and 33F	China	NCT06678620
24-valent polysaccharide conjugate vaccine	Jiangsu Kunli	18 June 2025	Phase I (adult, Older adults), completed	1, 2, 4, 5, 6A, 7F, 8, 9N, 9V, 10A, 11A, 14, 15B, 17F, 18C, 19A, 19F, 20, 22F, 23F, 33F	China	NCT07025876
Recombinant Pneumococcal Protein Vaccine PBPV	CanSinoBIO	26 April 2024	Phase I a/b (adult), completed	PspA	China	NCT04087460
31-valent polysaccharide conjugate vaccine (VAX-31)	Vaxcyte	16 December 2025; 26 January 2026	Phase III (adult); Phase II (infant), active, not recruiting	1, 2, 3, 4, 5, 6A, 6B, 7C, 7F, 8, 9N, 9V, 10A, 11A, 12F, 14, 15A, 15B, 16F, 17F, 18C, 19A, 19F, 20B, 22F, 23A, 23B, 23F, 31, 33F and 35B	U.S.	NCT07284654 (adult) NCT06720038 (infant)
25-valent polysaccharide conjugate vaccine (IVT PCV-25)	Inventprise Inc.	30 August 2023	Phase II (adult), completed	1, 2, 3, 4, 5, 6B, 6C, 7F, 8, 9N, 9V, 10A, 12F, 14, 15A, 15B, 16F, 18C, 19A, 19F, 22F, 23F, 24F, 33F, 35B	Canada	NCT05540028
24-valent polysaccharide conjugate vaccine (VAX-24)	Vaxcyte	11 September 2025; 18 April 2024; 19 June 2024	Phase II (infants, adults, Older adults), completed	1, 2, 3, 4, 5, 6A, 6B, 7F, 8, 9N, 9V, 10A, 11A, 12F, 14, 15B, 17F, 18C, 19A, 19F, 20, 22F, 23F and 33F	U.S.	NCT05844423; NCT05266456; NCT05297578
Whole-Inactivated pneumococcal vaccine (Gamma-PN3)	GPN Vaccines	5 September 2024	Phase I (adults, Older adults), completed	/	Australia	NCT05667740
Recombinant protein vaccine (MVX01)	Matrivax Research and Development Corporation	29 March 2024	Phase I (adults, Older adults), active, not recruiting	Ply CbpA	U.S.	NCT06337643

## Data Availability

No new data were created or analyzed in this study.

## References

[1] Li L., Ma J., Yu Z., Li M., Zhang W., Sun H. (2023). Epidemiological Characteristics and Antibiotic Resistance Mechanisms of Streptococcus Pneumoniae: An Updated Review. Microbiol. Res..

[2] Scelfo C., Menzella F., Fontana M., Ghidoni G., Galeone C., Facciolongo N.C. (2021). Pneumonia and Invasive Pneumococcal Diseases: The Role of Pneumococcal Conjugate Vaccine in the Era of Multi-Drug Resistance. Vaccines.

[3] Grant L.R., Meche A., McGrath L., Miles A., Alfred T., Yan Q., Chilson E. (2023). Risk of Pneumococcal Disease in US Adults by Age and Risk Profile. Open Forum Infect. Dis..

[4] Mohanty S., Cossrow N., Yu K.C., Ye G., White M., Gupta V. (2024). Clinical and Economic Burden of Invasive Pneumococcal Disease and Noninvasive All-Cause Pneumonia in Hospitalized US Adults: A Multicenter Analysis from 2015 to 2020. Int. J. Infect. Dis..

[5] Sari R.F., Fadilah F., Maladan Y., Sarassari R., Safari D. (2024). A Narrative Review of Genomic Characteristics, Serotype, Immunogenicity, and Vaccine Development of Streptococcus Pneumoniae Capsular Polysaccharide. Clin. Exp. Vaccine Res..

[6] Nelson A.L., Roche A.M., Gould J.M., Chim K., Ratner A.J., Weiser J.N. (2007). Capsule Enhances Pneumococcal Colonization by Limiting Mucus-Mediated Clearance. Infect. Immun..

[7] Narciso A.R., Dookie R., Nannapaneni P., Normark S., Henriques-Normark B. (2024). Streptococcus Pneumoniae Epidemiology, Pathogenesis and Control. Nat. Rev. Microbiol..

[8] Ali M.Q., Kohler T.P., Burchhardt G., Wüst A., Henck N., Bolsmann R., Voß F., Hammerschmidt S. (2021). Extracellular Pneumococcal Serine Proteases Affect Nasopharyngeal Colonization. Front. Cell. Infect. Microbiol..

[9] Mitsi E., Nikolaou E., Goncalves A., Blizard A., Hill H., Farrar M., Hyder-Wright A., Akeju O., Hamilton J., Howard A. (2024). RSV and Rhinovirus Increase Pneumococcal Carriage Acquisition and Density, Whereas Nasal Inflammation Is Associated with Bacterial Shedding. Cell Host Microbe.

[10] Bogaert D., de Groot R., Hermans P. (2004). Streptococcus Pneumoniae Colonisation: The Key to Pneumococcal Disease. Lancet Infect. Dis..

[11] Li J., Zhang J.-R. (2019). Phase Variation of Streptococcus Pneumoniae. Microbiol. Spectr..

[12] Oliver M.B., Swords W.E. (2020). Comparative Analysis of Streptococcus Pneumoniae Type I Restriction-Modification Loci: Variation in hsdS Gene Target Recognition Domains. Pathogens.

[13] Brissac T., Martínez E., Kruckow K.L., Riegler A.N., Ganaie F., Im H., Bakshi S., Arroyo-Diaz N.M., Spencer B.L., Saad J.S. (2021). Capsule Promotes Intracellular Survival and Vascular Endothelial Cell Translocation during Invasive Pneumococcal Disease. mBio.

[14] Kietzman C.C., Gao G., Mann B., Myers L., Tuomanen E.I. (2016). Dynamic Capsule Restructuring by the Main Pneumococcal Autolysin LytA in Response to the Epithelium. Nat. Commun..

[15] Neill D.R., Clarke T.B. (2025). The metabolic, microbial and immunological demands of pneumococcal colonisation. PLoS Pathog..

[16] Weight C.M., Venturini C., Pojar S., Jochems S.P., Reiné J., Nikolaou E., Solórzano C., Noursadeghi M., Brown J.S., Ferreira D.M. (2019). Microinvasion by Streptococcus Pneumoniae Induces Epithelial Innate Immunity during Colonisation at the Human Mucosal Surface. Nat. Commun..

[17] Attali C., Durmort C., Vernet T., Guilmi A.M.D. (2008). The Interaction of Streptococcus Pneumoniae with Plasmin Mediates Transmigration across Endothelial and Epithelial Monolayers by Intercellular Junction Cleavage. Infect. Immun..

[18] Nishimoto A.T., Rosch J.W., Tuomanen E.I. (2020). Pneumolysin: Pathogenesis and Therapeutic Target. Front. Microbiol..

[19] Hirst R.A., Sikand K.S., Rutman A., Mitchell T.J., Andrew P.W., O’Callaghan C. (2000). Relative Roles of Pneumolysin and Hydrogen Peroxide from Streptococcus pneumoniae in Inhibition of Ependymal Ciliary Beat Frequency. Infect. Immun..

[20] Palmer C.S., Kimmey J.M. (2022). Neutrophil Recruitment in Pneumococcal Pneumonia. Front. Cell. Infect. Microbiol..

[21] Lask A., Gutbier B., Kershaw O., Nouailles G., Gruber A.D., Müller-Redetzky H.C., Chackowicz S., Hamilton D.A., Van Slyke P., Witzenrath M. (2022). Adjunctive Therapy with the Tie2 Agonist Vasculotide Reduces Pulmonary Permeability in Streptococcus Pneumoniae Infected and Mechanically Ventilated Mice. Sci. Rep..

[22] Pereira J.M., Xu S., Leong J.M., Sousa S. (2022). The Yin and Yang of Pneumolysin During Pneumococcal Infection. Front. Immunol..

[23] Rosendahl A., Bergmann S., Hammerschmidt S., Goldmann O., Medina E. (2013). Lung Dendritic Cells Facilitate Extrapulmonary Bacterial Dissemination during Pneumococcal Pneumonia. Front. Cell. Infect. Microbiol..

[24] Rubins J.B., Janoff E.N. (1998). Pneumolysin: A Multifunctional Pneumococcal Virulence Factor. J. Lab. Clin. Med..

[25] Oliver M.B., Basu Roy A., Kumar R., Lefkowitz E.J., Swords W.E. (2017). Streptococcus pneumoniae TIGR4 Phase-Locked Opacity Variants Differ in Virulence Phenotypes. mSphere.

[26] Mukerji R., Mirza S., Roche A.M., Widener R.W., Croney C.M., Rhee D.-K., Weiser J.N., Szalai A.J., Briles D.E. (2012). Pneumococcal Surface Protein A Inhibits Complement Deposition on the Pneumococcal Surface by Competing with the Binding of C-Reactive Protein to Cell-Surface Phosphocholine. J. Immunol..

[27] Lu L., Ma Y., Zhang J.-R. (2006). Streptococcus Pneumoniae Recruits Complement Factor H through the Amino Terminus of CbpA. J. Biol. Chem..

[28] Shaper M., Hollingshead S.K., Benjamin W.H., Briles D.E. (2004). PspA Protects Streptococcus pneumoniae from Killing by Apolactoferrin, and Antibody to PspA Enhances Killing of Pneumococci by Apolactoferrin. Infect. Immun..

[29] Womack E., Alibayov B., Vidal J.E., Eichenbaum Z. (2023). Endogenously produced H2O2 is intimately involved in iron metabolism in Streptococcus pneumoniae. Microbiol. Spectr..

[30] Carreno D., Wanford J.J., Jasiunaite Z., Hames R.G., Chung W.Y., Dennison A.R., Straatman K., Martinez-Pomares L., Pareek M., Orihuela C.J. (2021). Splenic Macrophages as the Source of Bacteraemia during Pneumococcal Pneumonia. eBioMedicine.

[31] Brown A.O., Mann B., Gao G., Hankins J.S., Humann J., Giardina J., Faverio P., Restrepo M.I., Halade G.V., Mortensen E.M. (2014). Streptococcus Pneumoniae Translocates into the Myocardium and Forms Unique Microlesions That Disrupt Cardiac Function. PLoS Pathog..

[32] Shenoy A.T., Brissac T., Gilley R.P., Kumar N., Wang Y., Gonzalez-Juarbe N., Hinkle W.S., Daugherty S.C., Shetty A.C., Ott S. (2017). Streptococcus Pneumoniae in the Heart Subvert the Host Response through Biofilm-Mediated Resident Macrophage Killing. PLoS Pathog..

[33] Brissac T., Shenoy A.T., Patterson L.A., Orihuela C.J. (2017). Cell Invasion and Pyruvate Oxidase-Derived H2O2 Are Critical for Streptococcus Pneumoniae-Mediated Cardiomyocyte Killing. Infect. Immun..

[34] Liu Y.-C., Yu M.-M., Shou S.-T., Chai Y.-F. (2017). Sepsis-Induced Cardiomyopathy: Mechanisms and Treatments. Front. Immunol..

[35] Feldman C., Anderson R. (2020). Platelets and Their Role in the Pathogenesis of Cardiovascular Events in Patients With Community-Acquired Pneumonia. Front. Immunol..

[36] Iovino F., Engelen-Lee J.-Y., Brouwer M., van de Beek D., van der Ende A., Valls Seron M., Mellroth P., Muschiol S., Bergstrand J., Widengren J. (2017). pIgR and PECAM-1 Bind to Pneumococcal Adhesins RrgA and PspC Mediating Bacterial Brain Invasion. J. Exp. Med..

[37] Dutta R., Roy S. (2015). Chronic Morphine and HIV-1 Tat Promote Differential Central Nervous System Trafficking of CD3+ and Ly6C+ Immune Cells in a Murine Streptococcus Pneumoniae Infection Model. J. Neuroinflammation.

[38] Mahdi L.K., Wang H., der Hoek M.B.V., Paton J.C., Ogunniyi A.D. (2012). Identification of a Novel Pneumococcal Vaccine Antigen Preferentially Expressed during Meningitis in Mice. J. Clin. Investig..

[39] Barichello T., dos Santos I., Savi G.D., Florentino A.F., Silvestre C., Comim C.M., Feier G., Sachs D., Teixeira M.M., Teixeira A.L. (2009). Tumor Necrosis Factor Alpha (TNF-α) Levels in the Brain and Cerebrospinal Fluid after Meningitis Induced by *Streptococcus pneumoniae*. Neurosci. Lett..

[40] Famà A., Midiri A., Mancuso G., Biondo C., Lentini G., Galbo R., Giardina M.M., De Gaetano G.V., Romeo L., Teti G. (2020). Nucleic Acid-Sensing Toll-Like Receptors Play a Dominant Role in Innate Immune Recognition of Pneumococci. mBio.

[41] Lim J.H., Stirling B., Derry J., Koga T., Jono H., Woo C.-H., Xu H., Bourne P., Ha U.-H., Ishinaga H. (2007). Tumor Suppressor CYLD Regulates Acute Lung Injury in Lethal Streptococcus Pneumoniae Infections. Immunity.

[42] Licciardi P.V., Balloch A., Russell F.M., Nahm M.H., Mulholland K., Tang M.L.K. (2011). Pneumococcal Glycoconjugate Vaccines Produce Antibody Responses That Strongly Correlate with Function. Nat. Rev. Drug Discov..

[43] Pido-Lopez J., Kwok W.W., Mitchell T.J., Heyderman R.S., Williams N.A. (2011). Acquisition of Pneumococci Specific Effector and Regulatory Cd4+ T Cells Localising within Human Upper Respiratory-Tract Mucosal Lymphoid Tissue. PLoS Pathog..

[44] Ramos-Sevillano E., Ercoli G., Brown J.S. (2019). Mechanisms of Naturally Acquired Immunity to Streptococcus Pneumoniae. Front. Immunol..

[45] Syed S., Viazmina L., Mager R., Meri S., Haapasalo K. (2020). Streptococci and the Complement System: Interplay during Infection, Inflammation and Autoimmunity. FEBS Lett..

[46] Gil E., Noursadeghi M., Brown J.S. (2022). Streptococcus Pneumoniae Interactions with the Complement System. Front. Cell. Infect. Microbiol..

[47] Blom A.M., Bergmann S., Fulde M., Riesbeck K., Agarwal V. (2014). Streptococcus Pneumoniae Phosphoglycerate Kinase Is a Novel Complement Inhibitor Affecting the Membrane Attack Complex Formation. J. Biol. Chem..

[48] Andre G.O., Converso T.R., Politano W.R., Ferraz L.F.C., Ribeiro M.L., Leite L.C.C., Darrieux M. (2017). Role of Streptococcus Pneumoniae Proteins in Evasion of Complement-Mediated Immunity. Front. Microbiol..

[49] Hyams C., Camberlein E., Cohen J.M., Bax K., Brown J.S. (2010). The *Streptococcus pneumoniae* Capsule Inhibits Complement Activity and Neutrophil Phagocytosis by Multiple Mechanisms. Infect. Immun..

[50] Waz N.T., Milani B., Assoni L., Coelho G.R., Sciani J.M., Parisotto T., Ferraz L.F.C., Hakansson A.P., Converso T.R., Darrieux M. (2024). Pneumococcal Surface Protein A (PspA) Prevents Killing of Streptococcus Pneumoniae by Indolicidin. Sci. Rep..

[51] Dalia A.B., Standish A.J., Weiser J.N. (2010). Three Surface Exoglycosidases from Streptococcus Pneumoniae, NanA, BgaA, and StrH, Promote Resistance to Opsonophagocytic Killing by Human Neutrophils. Infect. Immun..

[52] Maestro B., Sanz J.M. (2016). Choline Binding Proteins from Streptococcus Pneumoniae: A Dual Role as Enzybiotics and Targets for the Design of New Antimicrobials. Antibiotics.

[53] Dalia A.B., Weiser J.N. (2011). Minimization of Bacterial Size Allows for Complement Evasion and Is Overcome by the Agglutinating Effect of Antibody. Cell Host Microbe.

[54] Briles D.E., Paton J.C., Mukerji R., Swiatlo E., Crain M.J. (2019). Pneumococcal Vaccines. Microbiol. Spectr..

[55] Guo X., Li J., Qiu J., Zhang R., Ren J., Huang Z., Li Z., Liang X., Lan F., Chen J. (2024). Persistence of Antibody to 23-Valent Pneumococcal Polysaccharide Vaccine: A 5-Year Prospective Follow-up Cohort Study. Expert Rev. Vaccines.

[56] Nielsen K.F., Nielsen L.B., Dalby T., Lomholt F.K., Slotved H.-C., Fuursted K., Harboe Z.B., Jørgensen C.S., Valentiner-Branth P. (2024). Follow-Up Study of Effectiveness of 23-Valent Pneumococcal Polysaccharide Vaccine Against All-Type and Serotype-Specific Invasive Pneumococcal Disease, Denmark. Emerg. Infect. Dis. J.-CDC.

[57] Li S., Liang H., Zhao S.-H., Yang X.-Y., Guo Z. (2023). Recent Progress in Pneumococcal Protein Vaccines. Front. Immunol..

[58] Feldman C. (2014). Review: Current and New Generation Pneumococcal Vaccines. Pneumococcal Vaccines.

[59] Djennad A., Ramsay M.E., Pebody R., Fry N.K., Sheppard C., Ladhani S.N., Andrews N.J. (2018). Effectiveness of 23-Valent Polysaccharide Pneumococcal Vaccine and Changes in Invasive Pneumococcal Disease Incidence from 2000 to 2017 in Those Aged 65 and Over in England and Wales. eClinicalMedicine.

[60] Weiser J.N., Ferreira D.M., Paton J.C. (2018). Streptococcus Pneumoniae: Transmission, Colonization and Invasion. Nat. Rev. Microbiol..

[61] Jochems S.P., Weiser J.N., Malley R., Ferreira D.M. (2017). The Immunological Mechanisms That Control Pneumococcal Carriage. PLoS Pathog..

[62] Berman-Rosa M., O’Donnell S., Barker M., Quach C. (2020). Efficacy and Effectiveness of t PCV-10 and PCV-13 Vaccines Against Invasive Pneumococcal Disease. Pediatrics.

[63] Zhang T., Zhang J., Shao X., Feng S., Xu X., Zheng B., Liu C., Dai Z., Jiang Q., Gessner B.D. (2021). Effectiveness of 13-Valent Pneumococcal Conjugate Vaccine against Community Acquired Pneumonia among Children in China, an Observational Cohort Study. Vaccine.

[64] Ryman J., Sachs J.R., Yee K.L., Banniettis N., Weaver J., Weiss T. (2024). Predicted Serotype-Specific Effectiveness of Pneumococcal Conjugate Vaccines V114 and PCV20 against Invasive Pneumococcal Disease in Children. Expert Rev. Vaccines.

[65] Pichichero M.E. (2013). Protein Carriers of Conjugate Vaccines. Hum. Vaccin. Immunother..

[66] Häfner S. (2020). Streptococcal Oddity: Article Highlight Based on “pspK Acquisition Contributes to the Loss of Capsule in Pneumococci: Molecular Characterisation of Non-Encapsulated Pneumococci” by Takeaki Wajima et Al. Microbes Infect..

[67] Levy C., Cohen R. (2024). Pneumococcal Conjugate Vaccine Schedule: 3+1, 2+1, or 1+1?. Lancet Child Adolesc. Health.

[68] Thindwa D., Shapiro E.D., Weinberger D.M. (2025). The Complex Landscape of Updated Pneumococcal Conjugate Vaccines. Open Forum Infect. Dis..

[69] Micoli F., Romano M.R., Carboni F., Adamo R., Berti F. (2023). Strengths and Weaknesses of Pneumococcal Conjugate Vaccines. Glycoconj. J..

[70] Smith K.J., Wateska A.R., Nowalk M.P., Lin C.J., Harrison L.H., Schaffner W., Zimmerman R.K. (2025). Cost-Effectiveness and Public Health Impact of 24-Valent Pneumococcal Conjugate Vaccine Compared With the Recommended Pneumococcal Vaccines in Older Adults. Am. J. Prev. Med..

[71] Fairman J., Agarwal P., Barbanel S., Behrens C., Berges A., Burky J., Davey P., Fernsten P., Grainger C., Guo S. (2021). Non-Clinical Immunological Comparison of a Next-Generation 24-Valent Pneumococcal Conjugate Vaccine (VAX-24) Using Site-Specific Carrier Protein Conjugation to the Current Standard of Care (PCV13 and PPV23). Vaccine.

[72] Behrens C., Fairman J., Agarwal P., Arulkumar S., Barbanel S., Bautista L., Berges A., Burky J., Davey P., Grainger C. (2021). 1047. Development of a Next Generation 30+ Valent Pneumococcal Conjugate Vaccine (VAX-XP) Using Site-Specific Carrier Protein Conjugation. Open Forum Infect. Dis..

[73] van der Put R.M.F., Metz B., Pieters R.J. (2023). Carriers and Antigens: New Developments in Glycoconjugate Vaccines. Vaccines.

[74] Thanawastien A., Cartee R.T., Griffin T.J., Killeen K.P., Mekalanos J.J. (2015). Conjugate-like Immunogens Produced as Protein Capsular Matrix Vaccines. Proc. Natl. Acad. Sci. USA.

[75] Kochetkov N.K., Nifant’ev N.E., Backinowsky L.V. (1987). Synthesis of the Capsular Polysaccharide of Streptococcus Pneumoniae Type 14. Tetrahedron.

[76] Gening M.L., Kurbatova E.A., Tsvetkov Y.E., Nifantiev N.E. (2015). Development of Approaches to a Third-Generation Carbohydrate-Conjugate Vaccine against *Streptococcus pneumoniae*: The Search for Optimal Oligosaccharide Ligands. Russ. Chem. Rev..

[77] Mettu R., Chen C.-Y., Wu C.-Y. (2020). Synthetic Carbohydrate-Based Vaccines: Challenges and Opportunities. J. Biomed. Sci..

[78] Biemans R., Micoli F., Romano M.R. (2020). Glycoconjugate Vaccines, Production and Characterization. Recent Trends in Carbohydrate Chemistry.

[79] Gopalakrishnan S., Jayapal P., John J. (2025). Pneumococcal Surface Proteins as Targets for Next-Generation Vaccines: Addressing the Challenges of Serotype Variation. Diagn. Microbiol. Infect. Dis..

[80] Malik T.M., Bakker K.M., Oidtman R.J., Sharomi O., Meleleo G., Nachbar R.B., Elbasha E.H. (2025). A dynamic transmission model for assessing the impact of pneumococcal vaccination in the United States. PLoS ONE.

[81] Pichichero M.E., Khan M.N., Xu Q. (2016). Next Generation Protein Based Streptococcus Pneumoniae Vaccines. Hum. Vaccines Immunother..

[82] Yuan Z.Q., Lv Z.Y., Gan H.Q., Xian M., Zhang K.X., Mai J.Y., Yu X.B., Wu Z.D. (2011). Intranasal Immunization with Autolysin (LytA) in Mice Model Induced Protection against Five Prevalent Streptococcus Pneumoniae Serotypes in China. Immunol. Res..

[83] Corsini B., Aguinagalde L., Ruiz S., Domenech M., Antequera M.L., Fenoll A., García P., García E., Yuste J. (2016). Immunization with LytB Protein of *Streptococcus pneumoniae* Activates Complement-Mediated Phagocytosis and Induces Protection against Pneumonia and Sepsis. Vaccine.

[84] García E. (2025). Structure, Function, and Regulation of LytA: The N-Acetylmuramoyl-l-Alanine Amidase Driving the “Suicidal Tendencies” of Streptococcus Pneumoniae—A Review. Microorganisms.

[85] Aceil J., Avci F.Y. (2022). Pneumococcal Surface Proteins as Virulence Factors, Immunogens, and Conserved Vaccine Targets. Front. Cell. Infect. Microbiol..

[86] Knupp-Pereira P.A., Marques N.T.C., Teixeira L.M., Póvoa H.C.C., Neves F.P.G. (2020). Prevalence of PspA Families and Pilus Islets among Streptococcus Pneumoniae Colonizing Children before and after Universal Use of Pneumococcal Conjugate Vaccines in Brazil. Braz. J. Microbiol..

[87] Xiao J., Liu B., Yin Y., Zhang X. (2024). Immunization with Recombinant Streptococcus Pneumoniae PgdA Protects Mice against Lung Invasion. Exp. Biol. Med..

[88] Chan J.M., Ramos-Sevillano E., Betts M., Wilson H.U., Weight C.M., Houhou-Ousalah A., Pollara G., Brown J.S., Heyderman R.S. (2024). Bacterial Surface Lipoproteins Mediate Epithelial Microinvasion by Streptococcus Pneumoniae. Infect. Immun..

[89] Paulikat A.D., Schwudke D., Hammerschmidt S., Voß F. (2024). Lipidation of Pneumococcal Proteins Enables Activation of Human Antigen-Presenting Cells and Initiation of an Adaptive Immune Response. Front. Immunol..

[90] Scott N.R., Mann B., Tuomanen E.I., Orihuela C.J. (2021). Multi-Valent Protein Hybrid Pneumococcal Vaccines: A Strategy for the Next Generation of Vaccines. Vaccines.

[91] Ginsburg A.S., Nahm M.H., Khambaty F.M., Alderson M.R. (2012). Issues and Challenges in the Development of Pneumococcal Protein Vaccines: A Two Day International Symposium. Expert Rev. Vaccines.

[92] Feng S., Xiong C., Wang G., Wang S., Jin G., Gu G. (2020). Exploration of Recombinant Fusion Proteins YAPO and YAPL as Carrier Proteins for Glycoconjugate Vaccine Design against Streptococcus Pneumoniae Infection. ACS Infect. Dis..

[93] Milani B., Santos T.W., Guerra M.E.S., Oliveira S., Goulart C., André G.O., Leite L.C.C., Converso T.R., Darrieux M. (2023). Fusion of PspA to detoxified pneumolysin enhances pneumococcal vaccine coverage. PLoS ONE.

[94] Liu X., Maele L.V., Matarazzo L., Soulard D., da Silva V.A.D., de Bakker V., Dénéréaz J., Bock F.P., Taschner M., Ou J. (2024). A Conserved Antigen Induces Respiratory Th17-Mediated Broad Serotype Protection against Pneumococcal Superinfection. Cell Host Microbe.

[95] Nguyen B., Tolia N.H. (2021). Protein-Based Antigen Presentation Platforms for Nanoparticle Vaccines. npj Vaccines.

[96] Jain S.S., Singh V.K., Kante R.K., Jana S.K., Patil R.H. (2024). Current Trends in Development and Manufacturing of Higher-Valent Pneumococcal Polysaccharide Conjugate Vaccine and Its Challenges. Biologicals.

[97] Gonçalves V.M. (2025). Novel Processes to Obtain Pneumococcal Surface Proteins for Vaccines. Appl. Microbiol. Biotechnol..

[98] Afshari E., Ahangari Cohan R., Shams Nosrati M.S., Mousavi S.F. (2023). Development of a Bivalent Protein-Based Vaccine Candidate against Invasive Pneumococcal Diseases Based on Novel Pneumococcal Surface Protein A in Combination with Pneumococcal Histidine Triad Protein D. Front. Immunol..

[99] Moreno A.T., Oliveira M.L.S., Ferreira D.M., Ho P.L., Darrieux M., Leite L.C.C., Ferreira J.M.C., Pimenta F.C., Andrade A.L.S.S., Miyaji E.N. (2010). Immunization of Mice with Single PspA Fragments Induces Antibodies Capable of Mediating Complement Deposition on Different Pneumococcal Strains and Cross-Protection. Clin. Vaccine Immunol..

[100] Wilson R., Cohen J.M., Reglinski M., Jose R.J., Chan W.Y., Marshall H., Vogel C., Gordon S., Goldblatt D., Petersen F.C. (2017). Naturally Acquired Human Immunity to Pneumococcus Is Dependent on Antibody to Protein Antigens. PLoS Pathog..

[101] Salha D., Szeto J., Myers L., Claus C., Sheung A., Tang M., Ljutic B., Hanwell D., Ogilvie K., Ming M. (2012). Neutralizing Antibodies Elicited by a Novel Detoxified Pneumolysin Derivative, PlyD1, Provide Protection against Both Pneumococcal Infection and Lung Injury. Infect. Immun..

[102] Ricci S., Janulczyk R., Gerlini A., Braione V., Colomba L., Iannelli F., Chiavolini D., Oggioni M.R., Björck L., Pozzi G. (2011). The Factor H-Binding Fragment of PspC as a Vaccine Antigen for the Induction of Protective Humoral Immunity against Experimental Pneumococcal Sepsis. Vaccine.

[103] Malley R., Trzcinski K., Srivastava A., Thompson C.M., Anderson P.W., Lipsitch M. (2005). CD4+ T Cells Mediate Antibody-Independent Acquired Immunity to Pneumococcal Colonization. Proc. Natl. Acad. Sci. USA.

[104] Musie E., Moore C.C., Martin E.N., Scheld W.M. (2014). Toll-Like Receptor 4 Stimulation before or after Streptococcus Pneumoniae Induced Sepsis Improves Survival and Is Dependent on T-Cells. PLoS ONE.

[105] Rodrigues T.C., Oliveira M.L.S., Soares-Schanoski A., Chavez-Rico S.L., Figueiredo D.B., Gonçalves V.M., Ferreira D.M., Kunda N.K., Saleem I.Y., Miyaji E.N. (2018). Mucosal Immunization with PspA (Pneumococcal Surface Protein A)-Adsorbed Nanoparticles Targeting the Lungs for Protection against Pneumococcal Infection. PLoS ONE.

[106] Balachandran P., Brooks-Walter A., Virolainen-Julkunen A., Hollingshead S.K., Briles D.E. (2002). Role of Pneumococcal Surface Protein C in Nasopharyngeal Carriage and Pneumonia and Its Ability To Elicit Protection against Carriage of Streptococcus Pneumoniae. Infect. Immun..

[107] Hermand P., Vandercammen A., Mertens E., Di Paolo E., Verlant V., Denoël P., Godfroid F. (2016). Preclinical Evaluation of a Chemically Detoxified Pneumolysin as Pneumococcal Vaccine Antigen. Hum. Vaccin. Immunother..

[108] Wang Y., Xia L., Wang G., Lu H., Wang H., Luo S., Zhang T., Gao S., Huang J., Min X. (2022). Subcutaneous Immunization with the Fusion Protein ΔA146Ply-SP0148 Confers Protection against *Streptococcus pneumoniae* Infection. Microb. Pathog..

[109] Ogunniyi A.D., Woodrow M.C., Poolman J.T., Paton J.C. (2001). Protection against Streptococcus Pneumoniae Elicited by Immunization with Pneumolysin and CbpA. Infect. Immun..

[110] da Silva M.A., Converso T.R., Gonçalves V.M., Leite L.C., Tanizaki M.M., Barazzone G.C. (2017). Conjugation of PspA4Pro with Capsular Streptococcus Pneumoniae Polysaccharide Serotype 14 Does Not Reduce the Induction of Cross-Reactive Antibodies. Clin. Vaccine Immunol..

[111] Perciani C.T., Barazzone G.C., Goulart C., Carvalho E., Cabrera-Crespo J., Gonçalves V.M., Leite L.C.C., Tanizaki M.M. (2013). Conjugation of Polysaccharide 6B from Streptococcus Pneumoniae with Pneumococcal Surface Protein A: PspA Conformation and Its Effect on the Immune Response. Clin. Vaccine Immunol..

[112] Kaplonek P., Yao L., Reppe K., Voß F., Kohler T., Ebner F., Schäfer A., Blohm U., Priegue P., Bräutigam M. (2021). A Semisynthetic Glycoconjugate Provides Expanded Cross-Serotype Protection against Streptococcus Pneumoniae. Vaccine.

[113] Chichili G.R., Smulders R., Santos V., Cywin B., Kovanda L., Van Sant C., Malinoski F., Sebastian S., Siber G., Malley R. (2022). Phase 1/2 Study of a Novel 24-Valent Pneumococcal Vaccine in Healthy Adults Aged 18 to 64 Years and in Older Adults Aged 65 to 85 Years. Vaccine.

[114] Rauch S., Jasny E., Schmidt K.E., Petsch B. (2018). New Vaccine Technologies to Combat Outbreak Situations. Front. Immunol..

[115] Mulligan M.J., Lyke K.E., Kitchin N., Absalon J., Gurtman A., Lockhart S., Neuzil K., Raabe V., Bailey R., Swanson K.A. (2020). Phase I/II Study of COVID-19 RNA Vaccine BNT162b1 in Adults. Nature.

[116] Kranz L.M., Diken M., Haas H., Kreiter S., Loquai C., Reuter K.C., Meng M., Fritz D., Vascotto F., Hefesha H. (2016). Systemic RNA Delivery to Dendritic Cells Exploits Antiviral Defence for Cancer Immunotherapy. Nature.

[117] Alberer M., Gnad-Vogt U., Hong H.S., Mehr K.T., Backert L., Finak G., Gottardo R., Bica M.A., Garofano A., Koch S.D. (2017). Safety and Immunogenicity of a mRNA Rabies Vaccine in Healthy Adults: An Open-Label, Non-Randomised, Prospective, First-in-Human Phase 1 Clinical Trial. Lancet.

[118] Feldman R.A., Fuhr R., Smolenov I., (Mick) Ribeiro A., Panther L., Watson M., Senn J.J., Smith M., Almarsson Ö., Pujar H.S. (2019). mRNA Vaccines against H10N8 and H7N9 Influenza Viruses of Pandemic Potential Are Immunogenic and Well Tolerated in Healthy Adults in Phase 1 Randomized Clinical Trials. Vaccine.

[119] Khlebnikova A., Kirshina A., Zakharova N., Ivanov R., Reshetnikov V. (2024). Current Progress in the Development of mRNA Vaccines Against Bacterial Infections. Int. J. Mol. Sci..

[120] Elia U., Levy Y., Cohen H., Zauberman A., Gur D., Hazan-Halevy I., Aftalion M., Benarroch S., Bar-Haim E., Redy-Keisar O. (2025). Novel Bivalent mRNA-LNP Vaccine for Highly Effective Protection against Pneumonic Plague. Adv. Sci..

[121] Harbison-Price N., Sebina I., Bolton R.A., Finn M., Cork A.J., Courtney I.G., Hancock S., Pelingon R., Richter J., Ericsson O. (2025). An mRNA Vaccine Encoding Five Conserved Group A Streptococcus Antigens. Nat. Commun..

[122] Xu S., Qi G., Liu R., Liu S., Wang A., Li W., Ruan K., Zhan L., Wang L., Fei C. (2025). Development of a Novel-Ionizable-Lipid-Based mRNA Vaccine for Broad Protection against *Streptococcus pneumoniae*. Mol. Ther. Nucleic Acids.

[123] Carsetti R., Quinti I. (2024). Editorial: IgA and Mucosal Immunity in Vaccinology and in Protection from Infection. Front. Cell. Infect. Microbiol..

[124] Klugman K.P. (2001). Efficacy of Pneumococcal Conjugate Vaccines and Their Effect on Carriage and Antimicrobial Resistance. Lancet Infect. Dis..

[125] Lynch J.M., Briles D.E., Metzger D.W. (2003). Increased Protection against Pneumococcal Disease by Mucosal Administration of Conjugate Vaccine plus Interleukin-12. Infect. Immun..

[126] Han F., Wang W., Shi M., Zhou H., Yao Y., Li C., Shang A. (2022). Outer Membrane Vesicles from Bacteria: Role and Potential Value in the Pathogenesis of Chronic Respiratory Diseases. Front. Cell Infect. Microbiol..

[127] Nakahashi-Ouchida R., Uchida Y., Yuki Y., Katakai Y., Yamanoue T., Ogawa H., Munesue Y., Nakano N., Hanari K., Miyazaki T. (2021). A Nanogel-Based Trivalent PspA Nasal Vaccine Protects Macaques from Intratracheal Challenge with Pneumococci. Vaccine.

[128] Wei P., Romanò C., Li C., Clergeaud G., Andresen T.L., Henriksen J.R., Hansen A.E., Clausen M.H. (2024). An Intranasal Cationic Liposomal Polysaccharide Vaccine Elicits Humoral Immune Responses against Streptococcus Pneumoniae. Commun. Biol..

[129] Rodrigues T.C., Figueiredo D.B., Gonçalves V.M., Kaneko K., Saleem I.Y., Miyaji E.N. (2024). Liposome-Based Dry Powder Vaccine Immunization Targeting the Lungs Induces Broad Protection against Pneumococcus. J. Control. Release.

[130] Ortiz Moyano R., Raya Tonetti F., Elean M., Imamura Y., Fukuyama K., Suda Y., Melnikov V., Suvorov A., Vizoso-Pinto M.G., Kitazawa H. (2024). Bacterium-like Particles from Corynebacterium Pseudodiphtheriticum as Mucosal Adjuvant for the Development of Pneumococcal Vaccines. Vaccines.

[131] Lee C.-J., Lee L.H., Gu X.-X. (2005). Mucosal Immunity Induced by Pneumococcal Glycoconjugate. Crit. Rev. Microbiol..

[132] Kataoka K., Fukuyama Y., Briles D.E., Miyake T., Fujihashi K. (2017). Dendritic Cell-Targeting DNA-Based Nasal Adjuvants for Protective Mucosal Immunity to Streptococcus Pneumoniae. Microbiol. Immunol..

[133] Fujimoto K., Uematsu S. (2020). Development of Prime–Boost-Type next-Generation Mucosal Vaccines. Int. Immunol..

[134] Chen C., Liceras F.C., Flasche S., Sidharta S., Yoong J., Sundaram N., Jit M. (2019). Effect and Cost-Effectiveness of Pneumococcal Conjugate Vaccination: A Global Modelling Analysis. Lancet Glob. Health.

[135] Whitney C.G., Goldblatt D., O’Brien K.L. (2014). Dosing Schedules for Pneumococcal Conjugate Vaccine. Pediatr. Infect. Dis. J..

